# Acceptability and feasibility of the standardized direct observation assessment tool in the emergency department in Qatar

**DOI:** 10.5116/ijme.5a2e.7e16

**Published:** 2017-12-21

**Authors:** Saleem Farook, Sohaib Chaudhry, Baha Al Kahlout, Furqan B Irfan, Sameer Pathan

**Affiliations:** 1 Department of Emergency Medicine, Hamad General Hospital, Hamad Medical Corporation, Doha, Qatar

**Keywords:** Acceptability, feasibility, standardized, direct observation assessment tool, emergency department, Qatar

## Introduction

Workplace-based Assessments (WBAs) are important tools designed to assess the performance of residents in a clinical setting. The Standardized Direct Observation Tool (SDOT) is a WBA tool used by emergency medicine (EM) residency programs to assess the performance of residents. The SDOT, originally designed for use in the United States (US) residency programs, was implemented in other international programs as a part of their accreditation by the Accreditation Council for Graduate Medical Education-International (ACGME-I).^1^ Along with the many foundational requirements, the ACGME-I sets out the six core competencies of medical knowledge, patient care, professionalism, interpersonal communication skills, practice-based learning, and system-based practice. The SDOT is designed to assess these competencies.

Studies on the SDOT have focused on its reliability, but have not assessed its feasibility and acceptability to practitioner and institutions.^2^^,^^3^ The busy clinical practice environment of an emergency department (ED) presents numerous challenges which could adversely impact the implementation of the SDOT. The resource implications of time spent in conducting and documenting, the potential cost of the physicians' time and other administrative variables need consideration.

The EM residency program (EMRP) in Qatar is based at one of the busiest EDs in the world, with an annual attendance of over four hundred thousand patients. The SDOT was introduced to the EMRP in 2013 as a part of ACGME-I accreditation. The EMRP had a rich mix of approximately 40 residents and 100 faculty physicians drawn from international training and cultural backgrounds for those who the concept of WBA was unfamiliar. In its early phase, the implementation of the SDOT was mitigated by several feasibility and acceptability issues. Therefore, we embarked on an evaluation of SDOT use in the ED with an intention to make subsequent improvements to its implementation within the program. The aim of the paper is to explain our experiences and the lessons learned of the acceptability and feasibility of the SDOT in Qatar.

### Acceptability and feasibility issues

A WBA tool, like other tools, should be reliable, valid, feasible and acceptable to learners and assessment leads. It should be also effective in relation to its cost, and it should have educational power.^4^^,^^5^ To examine the acceptability and feasibility of the SDOT in Qatar, we identified the time available for both the faculty and the residents as a significant factor.  An unusually long time taken to complete the SDOT may indicate a lack of practicality of the tool. Conversely, insufficient time spent on feedback may limit its usefulness as a learning tool. There are no recommendations in the literature for the time taken to complete the SDOT. However, studies of other WBA tools in EM offer clues. In a study of the Mini-Clinical Examination tool (Mini-CEX) by interns in EM, the median time of 19 minutes for observation and 10 minutes for feedback were reported.^6^ In the milieu of a busy ED, time to undertake the SDOT could be a surmountable issue by ensuring adequate staffing levels and appropriate workload for faculty and residents.

The SDOT tool, consisting of a 34 item-checklist of competencies, is ambitious in its educational intention and resource intensiveness. To be deployed within a busy ED, the tool needs to be manageable in length and content. Otherwise, some users might choose to skip through the tool or delay the filling of the tool after the clinical interaction, leading to compromising the proposed advantages of immediate feedback. During the implementation of the SDOT, we found some issues in the rate of completion of the tools, highlighting the length of the tool as a potential area for improvement by making it more concise.

In addition, we noted that the introduction of a new assessment tool may reveal acceptability issues due to possible error variances which can distort the interpretation of assessment results. Specifically, we were concerned that the complex educational language of the SDOT might cause potential confusion to our multicultural faculty members and residents for whom English was not the first language. Furthermore, most of them were previously unfamiliar with the tool. Therefore, we undertook training workshops to improve their knowledge of the tool, which also provided them with the opportunity to enhance their understanding of a new concept in graduate medical assessment. With the help of these interventions, we found that the SDOT was received with an overall positive response to its perceived usefulness.

### Implications for graduate medical education

Feasibility issues such as the time to conduct assessments, the busy nature of clinical work and lack of training, while not unique to EM, are brought into sharp focus when administrating a new assessment tool such as the SDOT. A major systematic review of WBAs identified areas of similar concern with insufficient time, inadequate training, and negative user perceptions.^7^ As service pressures grow in busy specialties like EM, the much-needed balance of education and service may be tilted against the use of WBA tools at the clinical workplace. In this context, the targeted improvement programs such as the Clinical Learning Environment Review (CLER) program^8^ may provide an opportunity to review useful data about time and other factors influencing the practical implementation of WBAs. For example, calculations commonly proposed for the number of patients to be seen in an hour may need to be revised to take account of the extra time needed for performing the WBAs. Further studies to elicit true observational data such as time, clinical setting, and other logistics to help with the implementation of WBA may be worthwhile.

Improving the acceptability of new WBAs by faculty members and residents may be key to their implementation. Experienced faculty physicians, who themselves had been assessed using historical 'pass or fail' system of traditional examinations, might not readily endorse the use of these formative assessment tools based in the clinical workplace. Residents, not previously inducted into the ethos of WBAs, might not fully understand their intended use as tools for feedback and formative development. For these reasons, training of both faculty members and residents would be crucial for effective implementation of WBAs, including video-based training.^9^ An area for potential research is to assess the impact of training on the effectiveness of WBAs in EM, especially in Arabian countries.

## Conclusions

Predictable issues may arise with the implementation of the SDOT in EM programs, and indeed this could be said for any WBA, due to the busy clinical setting and the associated time pressures of ED clinical practice. Despite these logistical issues, the SDOT remains a potentially useful tool for assessing the six ACGME-I competencies in EM in Qatar. We suggest due consideration may be given to improving the acceptability and feasibility of this tool through appropriate time allocation and training of faculty, in addition to adapting the length of the form to make it more user-friendly. Our experience may be relevant to other international EM residency programs aspiring to implement the SDOT. However, further work needs to be done to establish the acceptability and feasibility of the SDOT in such programs.

### Conflict of Interest

The authors declare that they have no conflict of interest.

## References

[1] ACGME. Program requirements for graduate medical education. 2011 [cited 29 October 2017]; Available from: http://www.acgmei.org/Portals/0/FoundInternational03312016.pdf.

[2] Shayne P, Gallahue F, Rinnert S, Anderson CL, Hern G, Katz E (2006). Reliability of a core competency checklist assessment in the emergency department: the standardized direct observation assessment tool.. Acad Emerg Med.

[3] LaMantia J, Kane B, Yarris L, Tadros A, Ward MF, Lesser M, Shayne P (2009). Real-time inter-rater reliability of the Council of Emergency Medicine residency directors standardized direct observation assessment tool.. Acad Emerg Med.

[4] Van Der Vleuten CP (1996). The assessment of professional competence: developments, research and practical implications.. Adv Health Sci Educ Theory Pract.

[5] Academy of Medical Royal Colleges. Improving assessment. Academy of Medical Royal Colleges. 2009 [cited 29 October 2017]; Available from: http://www.aomrc.org.uk/doc_view/49-improving assessment.

[6] Brazil V, Ratcliffe L, Zhang J, Davin L (2012). Mini-CEX as a workplace-based assessment tool for interns in an emergency department--does cost outweigh value?. Med Teach.

[7] Massie J, Ali JM (2016). Workplace-based assessment: a review of user perceptions and strategies to address the identified shortcomings.. Adv Health Sci Educ Theory Pract.

[8] Weiss KB, Wagner R, Nasca TJ (2012). Development, testing, and implementation of the ACGME clinical learning environment review (CLER) program.. J Grad Med Educ.

[9] Kane KE, Weaver KR, Barr GC, Bonfante G, Bendock NL, Berry BM, Goren-Garcia SL, Lewbart MB, Raines AL, Smeriglio G, Kane BG (2017). Standardized direct observation assessment tool: using a training video.. J Emerg Med.

